# Neural Mechanisms Involved in Social Conformity and Psychopathic Traits: Prediction Errors, Reward Processing and Saliency

**DOI:** 10.3389/fnbeh.2019.00160

**Published:** 2019-07-16

**Authors:** Sandy Overgaauw, Myrthe Jansen, Naomi J. Korbee, Ellen R. A. de Bruijn

**Affiliations:** ^1^Department of Clinical Psychology, Institute of Psychology, Leiden University, Leiden, Netherlands; ^2^Leiden Institute for Brain and Cognition (LIBC), Leiden University, Leiden, Netherlands

**Keywords:** social conformity, social reward, psychopathic traits, amygdala, fMRI

## Abstract

Aligning behavior in favor of group norms, i.e., social conformity, can help to successfully adapt to uncertain environments and may result in social approval. This may lead to enhanced feelings of belongingness and is found to be associated with reward-related activations in the brain. Individuals high on psychopathic traits violate group norms regularly. Yet, it is unclear how psychopathic traits are related to neural mechanisms involved in social conformity. This functional magnetic resonance imaging (fMRI) study includes 42 healthy females scoring low or high on the Psychopathic Personality Inventory questionnaire (PPI). Participants were asked to rate the trustworthiness of 120 faces while lying in the scanner. After rating each face, participants were presented with the group rating of European students. In an unanticipated second part participants rated all faces again, allowing us to focus on two main contrasts: (1) “Social conflict”: group opinion in conflict with the participant’s rating vs. group opinion aligned with participant rating; and (2) “Conformity”: conflict trials followed by conformity vs. conflict trials followed by non-conformity. Behaviorally, the two groups showed similar conformity behavior. fMRI results showed that both groups activated the nucleus accumbens (NAc) following alignment, suggesting the central role of prediction errors and reward. The data also showed a significant interaction between group and conformity in the amygdala. Following conflicts, females scoring low on psychopathic traits showed a trend in enhanced amygdala activation for conformity relative to non-conformity. Additionally, results showed a trend significant group effect for non-conformity. Females scoring high on psychopathic traits showed more activation for non-conformity compared to females scoring low on psychopathic traits, suggesting altered emotional salience of experiencing conflict depending on psychopathic traits. Taken together, these results support the importance of investigating the role of relevant traits in adaptive behavior when facing uncertain social situations and the neural mechanisms involved in this process.

## Introduction

People regularly change their opinion and behavior in order to align with group norms. For example, when you stop talking to your friend because the people around you fall silent complying with 2 min of silence on the Dutch day of remembrance. Acquiring knowledge by observing how other people behave or how they make decisions can help in making adequate adjustments to specific circumstances (Van de Waal et al., 2013), but it also helps in gaining social approval of others (Bond and Smith, 1996). This phenomenon of aligning behavior in favor of group norms is called social conformity (for a review, see Cialdini and Goldstein, 2004).

Adopting the opinion or behavior of a group can facilitate successful adaptation to uncertain social environments and may result in social approval leading to greater feelings of belongingness (Cialdini and Goldstein, 2004). Previous studies already showed that aligning with group norms can result in the involvement of the nucleus accumbens (NAc), demonstrating that adapting your behavior adequately and according to the social norms is associated with feelings of (social) reward (Campbell-Meiklejohn et al., 2010; Nook and Zaki, 2015). However, not everyone seems to care as much about adhering to social norms. Previous studies focusing on incarcerated individuals scoring high on psychopathic traits demonstrated that these individuals show a persistent violation of social norms and expectations (Hare et al., 1991; Lilienfeld and Andrews, 1996). They also suffer from affective and interpersonal deficits such as a lack of empathy, guilt and remorse, shallow emotions, and manipulative behavior (Hare et al., 1991; Lilienfeld and Andrews, 1996). However, prior studies did find that psychopaths experience social approval as rewarding. Nonetheless, their motivation seems to be different, as they might see social approval as a conformation that they successfully manipulated others and that they can use others for personal gain (Foulkes et al., 2014a). Yet, it is unclear how individual differences in psychopathic traits within a non-clinical sample are related to the neural mechanisms involved in social conformity. Therefore, the current study will investigate the neural mechanisms involved in conflicting feedback situations in which an individual opinion deviates from that of a group. Additionally, this study aims to unravel the neural mechanisms involved in aligning with group norms—i.e., social conformity—in subjects scoring low or high on psychopathic traits in order to test for group differences.

Social conformity was first demonstrated experimentally by Asch (1951) and has become a well-established and well-studied phenomenon over the years (for a review, see Stallen and Sanfey, 2015). Yet, only more recently neuroimaging studies have started to investigate the neural mechanisms of social conformity (for a review, see Schnuerch and Gibbons, 2014). Klucharev et al. (2009) designed a social conformity paradigm, in which participants were asked to rate the attractiveness of female faces, and subsequently were presented with a group norm (the average rating of European students), which could be either in conflict or in alignment with their own initial opinion. In order to detect whether participants would conform to the (simulated) attractiveness norm of European students, participants were asked to rate the same faces again (behaviorally) after they finished the functional magnetic resonance imaging (fMRI) session. Using this conformity paradigm, conflict with group opinion has been found to elicit prediction error signals in the rostral cingulate zone (RCZ) and the NAc. Additionally, Klucharev et al. (2009) showed that the neural signals in RCZ (activation) and NAc (deactivation) predicted participant’s subsequent decision to conform to the group.

Evidence thus suggests that conformity is based on neural reinforcement-learning mechanisms, meaning that conforming behavior is reinforced by neural signals evoked by the conflict and alignment of own opinion with group norms (Schnuerch and Gibbons, 2015). These strong mechanisms are crucial for our motivation to be compliant with social norms and are essential for our survival (Cialdini and Goldstein, 2004). Posterior medial frontal cortex (including RCZ) and NAc play an important role in reinforcement learning (Holroyd and Coles, 2002; O’Doherty et al., 2004; Ridderinkhof et al., 2004; Klucharev et al., 2011), but also in the detection of errors and conflict, as well as in monitoring unfavorable outcomes (Ridderinkhof et al., 2004; de Bruijn et al., 2009; Radke et al., 2011). The NAc is also thought to play a central role in signaling errors in reward prediction (O’Doherty et al., 2004), and is implicated in the anticipation (Harsay et al., 2011) and experience of reward (O’Doherty et al., 2004).

When being part of a group, the implicit social rule to comply with the opinion of the majority is very common. Interestingly, when experiencing a conflict with a group norm, several processes could play a role: (1) detection of conflict; (2) reinforcement-learning; (3) monitoring of negative outcomes; but also (4) emotions elicited in response to a conflict. For example, in the study by Berns et al. (2005), participants performed a mental rotation task, either together with a group of peers or with a computer. Group and computer responses were manipulated so that the incorrect answer was given in one-third of the trials to induce conformity behavior. The authors demonstrated that when participants were in conflict with the group vs. the computer, amygdala activation was found. This brain area is involved in emotional learning, most notably in aversive learning (Berns et al., 2005; Belova et al., 2008; Roesch et al., 2010; Li et al., 2011; Klavir et al., 2013). As this activation was unique for situations in which participants were interacting with humans, Berns et al. (2005) suggested that this activation likely reflected the aversiveness and emotional salience of experiencing a social conflict. This outcome may, therefore, reflect an emotional route towards conformity, as amygdala activation during conflict with the group could signal the presence of an aversive event that one wants to avoid in the future.

How psychopathic traits are associated with neural mechanisms involved in social (non)conformity is yet unclear. We know from literature examining psychopaths in the criminal justice system that their failure to conform to social norms is often one of the reasons leading to their incarceration (Hare and Neumann, 2009). However, there are to our knowledge no studies that investigated the relationship between psychopathy or psychopathic traits and brain activation when experiencing a social conflict. There is, nonetheless, some indirect evidence linking psychopathic traits to disturbed prediction error signaling in the brain. The prediction error signal in the RCZ is thought to be reflected by an event-related component called the feedback-related negativity (FRN; Ridderinkhof et al., 2004). The FRN has been found to predict behavioral adjustments, including adjustments in conformity paradigms involving, for example, line- and facial judgment tasks (Chen et al., 2012; Schnuerch and Gibbons, 2015). Prior electrophysiological studies have linked psychopathy-related constructs to decreased amplitudes of the FRN (Schulreich et al., 2013; Leno et al., 2016; Schulreich, 2016). Other studies, however, failed to find an association between the FRN and psychopathic traits (von Borries et al., 2010; Varlamov et al., 2011; Salim et al., 2015). Although findings are mixed, there is evidence for aberrant prediction error signaling.

Apart from EEG studies showing indirect evidence for the link between psychopathic traits and aberrant prediction error signaling on a neural level, there are also some fMRI studies supporting this. A fMRI study performed by White et al. (2013) suggested that psychopathic traits might be related to impaired prediction error signaling in the NAc. Their results showed that youth with conduct and oppositional defiant disorder showed reduced responsiveness to positive prediction errors (unexpected reward) and increased responsiveness to negative prediction errors (unexpected omission of reward) within the NAc while receiving feedback in a passive avoidance task. Moreover, Geurts et al. (2016) studied the neural mechanisms underlying reward expectations in psychopathic criminals and showed enhanced reward-related connectivity between the striatum (part of the NAc) and the dorsomedial prefrontal cortex—a region involved in cognitive control—during reward vs. no reward expectancy compared to healthy controls. Taken together, these studies additionally suggest that psychopathic traits could be related to disturbed prediction error signaling and reward expectancy in the RCZ and the NAc during social conflict in a social conformity task.

Another region involved in social conformity, which has repeatedly been found to show altered activations in individuals scoring high on psychopathic traits, is the amygdala. Several fMRI studies investigating social functioning in incarcerated psychopaths showed decreased amygdala and rostral anterior cingulate cortex (rACC) activation when facing immoral situations (Glenn et al., 2009; Harenski et al., 2010, 2014; Carré et al., 2013). The study by Carré et al. (2013) focused on psychopathic traits in community samples and how these traits related to brain activation following social cues. Evidence has been found for distinct neural activity while observing angry faces. Exclusively in females, Carré et al. (2013) found a positive association between ventral striatum (part of NAc) activity and coldheartedness, whereas exclusively in males they found a positive association between amygdala and impulsivity. Overall, these findings indicate that psychopathy might be associated with disturbances in neural areas thought to be involved in social conformity (i.e., RCZ, NAc, and amygdala). However, it remains unclear how psychopathic traits relate to neural activity while showing (non)conformity behavior following a social conflict. In order to test this, we used the social conformity paradigm designed by Klucharev et al. (2009), while focusing on the trustworthiness of female faces in line with Campbell-Meiklejohn et al. (2010).

The trustworthiness of someone’s face is important for deciding whether to approach or to avoid this person, especially without additional contextual information (for example when only seeing a picture of a neutral face; Todorov, 2008). Relying on the group norm about whether or not to trust a person could help in preventing threatening situations. Previous studies including male violent offenders have demonstrated a lack of threat-avoiding abilities when facing social threat (Louise von Borries et al., 2012; Vieira et al., 2014), which has been found to be related to amygdala dysfunction (Kennedy et al., 2009). This distorted ability in approach/avoidance tendency could influence the extent to which individuals scoring high on psychopathic traits will conform to the group norm regarding the trustworthiness of faces, based on their altered perception. Additionally, a prior study found that females high on psychopathy reported lower levels of trust in response to a cooperative situation (Rilling et al., 2007), which could again influence their social conformity behavior.

In the current study, we hypothesized that females scoring high on psychopathic traits would show reduced conformity to a normative group opinion compared to females scoring low on these traits. Studying females in the context of conformity behavior is relevant as several prior studies have shown that females tend to conform more than males (Cooper, 1979; Eagly and Carli, 1981; Bond and Smith, 1996). In addition, our decision to only include women was also based on the findings of several prior studies that demonstrated significant higher psychopathic trait scores in males compared to females in community samples (Cale and Lilienfeld, 2002; Hemphälä and Tengström, 2010; Berkout et al., 2011). The present study addresses gaps in current knowledge on psychopathic traits by focusing on performance monitoring in a social context and by comparing the top 25% and bottom 25% of self-reported psychopathic traits (in line with Shao and Lee, 2017) in healthy female volunteers.

Although we know from previous studies that psychopathy is associated with norm-violating behavior and reduced empathic concern, which could lead to less conformity behavior (Kiehl and Hoffman, 2011; Seara-Cardoso et al., 2012; Foulkes et al., 2014b), we also take into account the possibility that females scoring high on psychopathic traits show no difference in conformity behavior. Perhaps they also experience social approval as rewarding, although with a different motivation (Foulkes et al., 2014a). On a neural level, we hypothesized, based on the findings by Klucharev et al. (2009), Campbell-Meiklejohn et al. (2010) and Berns et al. (2005), that overall conflict with group opinion would result in activation in the RCZ, amygdala, and deactivation in the NAc. Moreover, if activity in these regions predicts a participant’s subsequent decision to conform to group opinion following a conflicting situation, then activation should be stronger in those trials where conflict with the group led to conformity than in trials where social conflict did not result in conformity (Klucharev et al., 2009). Furthermore, based on the evidence summarized above suggesting impaired prediction error signaling in the RCZ and NAc in individuals scoring high on psychopathic traits in several reinforcement learning and error monitoring paradigms (Pfabigan et al., 2011; Schulreich et al., 2013; White et al., 2013; Leno et al., 2016), and based on the abundance of studies showing an association between psychopathy and amygdala dysfunction (Blair, 2008, 2013), we hypothesized that activity in the RCZ, NAc and amygdala would be modulated by individual differences in psychopathic traits in a social conformity task. In order to test this, we focused on two contrasts: (1) the “Social conflict” contrast: group opinion in conflict with participant rating vs. group opinion aligned with participant rating; and (2) the “Conformity” contrast: conflict trials followed by conformity vs. conflict trials followed by no conformity.

## Materials and Methods

### Participants

The 42 participants that were included in this study (*M* = 19.85 years, SD = 1.34) were all female, right-handed, fluent in Dutch, and without neurological or psychiatric disorders; see Table 1 for an overview of the group characteristics. To recruit females scoring low or high on psychopathic traits, we created a large pool of potential participants through advertisements on social media and the Leiden University Research Participation System called SONA. Participants completed a battery of questionnaires including the validated Dutch translation of the short-form of the Psychopathic Personality Inventory (PPI-SF: Tonnaer et al., 2013; see “Measures” section). We selected females scoring low (25th percentile) or high (75th percentile) on the PPI-SF from a total of 1,057 female adults. Participants completed the experiment for course credits or monetary compensation and provided written informed consent. The study was approved by the Institutional Review Board of the University Medical Center and conducted in accordance with the Declaration of Helsinki.

**Table 1:** Group characteristics of females scoring low and high on psychopathic traits (means and SDs).

	Low PPI (*N* = 22)	High PPI (*N* = 20)	*p*-value
Age	19.97 (1.48)	19.71 (1.20)	0.536
PPI-SF
Total	162.86 (12.76)	229.25 (10.22)	<0.001
Machievellian egocentricity	25.68 (4.47)	39.50 (5.91)	<0.001
Social potency	31.59 (6.88)	47.05 (7.05)	<0.001
Fearlessness	19.09 (5.02)	29.35 (4.97)	<0.001
Coldheartedness	18.77 (4.48)	25.55 (10.35)	0.012
Impulsive non-conformity	19.00 (2.89)	26.30 (4.58)	<0.001
Externalization of guilt	15.36 (3.31)	21.85 (5.25)	<0.001
Carefree non-planfulness	19.36 (5.23)	23.40 (7.65)	0.051
Stress immunity	14.00 (4.26)	16.25 (4.55)	0.106

*PPI-SF, Psychopathic Personality Inventory Short-Form*.

### Measures

To assess psychopathic traits, participants completed the PPI-SF (Tonnaer et al., 2013). The 100-item PPI-SF is answered on a 4-point Likert scale (1-*false* and 4-*true*) and contains eight subscales: (1) machiavellian egocentricity (ruthlessness and narcissism in interpersonal functioning); (2) social potency (perceived ability to influence and manipulate others); (3) coldheartedness (callousness, guiltlessness, and unsentimentality); (4) carefree nonplanfulness (attitude of indifference in planning one’s actions); (5) fearlessness (absence of anticipatory anxiety concerning harm and risk-taking behavior); (6) blame externalization (externalizing and rationalizing misbehavior); (7) impulsive nonconformity (reckless lack of concern regarding social mores); and (8) stress immunity (absence of emotional reactions to anxiety-provoking events).

### Stimuli

We used a validated set of 120 digital photos of European females previously employed by Klucharev et al. (2009) and in accordance to Campbell-Meiklejohn et al. (2010). In our study, we focused on trustworthiness ratings in contrast to the attractiveness ratings used by Klucharev et al. (2009), but in line with Campbell-Meiklejohn et al. (2010). Trustworthiness judgments are positively correlated with judgments of attractiveness (Todorov et al., 2008), which means that, similar to attractiveness judgments, cross-gender ratings of trustworthiness might be associated with mate selection. By using only female faces and female participants, this gender bias was avoided.

### Experimental Paradigm

**Figure 1:**
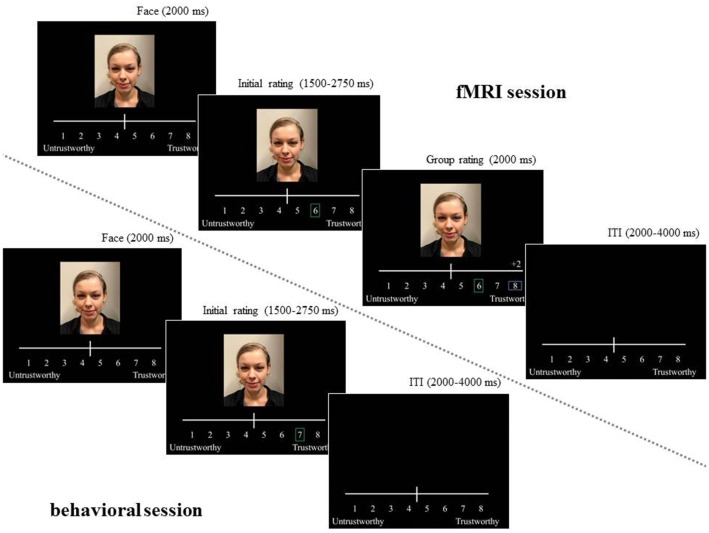
Example of trial during functional magnetic resonance imaging (fMRI) and behavioral session of the Social Conformity Task. During the fMRI session, participants were asked to rate the trustworthiness of female faces on a scale from 1 (untrustworthy) to 8 (trustworthy). The participant’s rating was visualized by a green rectangle frame that was followed by a presentation of the group rating of European students (blue horizontal rectangle frame). The difference in rating was presented above the “average European student” group rating (−3, −2, 0, +2, or +3). During the behavioral session (±20 min after fMRI session), participants were asked to rate the same faces again. This time, participants did not see the opinion of the European students. The subject in the figure gave permission to use her image by providing written informed consent.

Participants were told that they were taking part in a large scale European study called EuroTrust that aims to investigate how students at European universities perceive human trustworthiness. The logos of the “participating” European universities were included at the bottom of the instruction screen. During the fMRI session, participants rated the trustworthiness of 120 female faces on a scale from 1 (untrustworthy) to 8 (trustworthy; see Figure 1). Participants were able to answer as soon as the face was presented, but only after 2 s the participant’s rating was visualized on the screen. The participant’s decision was indicated by a green vertical rectangle frame (jittered between 1,500 and 2,750 ms). Then, during a 2 s period, the participant was presented with the group rating of the “average European student” of the same face indicated by a blue horizontal rectangle frame. The difference between the participant’s rating and the “average European student” group rating was also presented above the scale and could be: −3, −2, 0, +2, or +3 points. The inter-trial interval was jittered between 2 and 4 s. Participants were informed that the “average European student” group ratings that matched their own rating within a 1 point range were perceived as no difference (i.e., 0 points). The task was programmed so that the “average European student” group rating agreed with the participant’s rating in 33 percent of the trials (= 40 trials), whereas in 67 percent of the trials the “average European student” group ratings were either above or below participant’s rating by 2 or 3 points (each 20 trials).

In an unanticipated second part, about 20 min after the fMRI session, participants rated all 120 faces again (in a newly randomized order), but this time without presentation of group feedback and outside of the scanner (see Figure 1). At the end of the experiment, participants received both oral and written questions about their responses on the task to check whether the manipulation worked as intended.

Alongside this study, we performed a behavioral control study. Participants in the control study (*N* = 32) also performed the task twice. However, in their version of the task, participants were simply instructed to rate the faces without group opinion being mentioned or presented. This was done in order to control for the effect of regression to the mean (RTM; Schnuerch et al., 2015); see behavioral data analyses for a complete description.

### Behavioral Data Analyses

Prior to the analyses, we mean-centered all ratings by subtracting the mean of all trustworthiness ratings from each separate rating. Subsequently, we subtracted the first session trustworthiness ratings from the second session ratings to obtain a rating change score for each item. We followed the approach by Schnuerch et al. (2015) of adding a control group in order to assess and rule out the effect of RTM. RTM is the phenomenon that extreme values at first measurement tend to approach the mean on subsequent measurement (Barnett et al., 2005). By using a control group, which merely rated all images twice without being presented with group opinion (i.e., the social-influence manipulation), we could assess the isolated effect of initial ratings on subsequent rating changes. From the control group, a hierarchical linear model was derived that allowed to predict rating changes on the basis of initial ratings. In line with Schnuerch et al. (2015), a random-coefficient model was fitted using R packages lme4 (Bates et al., 2015) and lmerTest (Kuznetsova et al., 2017), which uses Satterthwaite’s degrees of freedom method. Subsequently, this model was applied to the experimental group in order to estimate the expected rating change caused by the level of the initial rating (i.e., RTM). This RTM estimate was then used to obtain a corrected rating-change estimate per item for the experimental group that captured only the influence of group deviation. A 3-level factor “social influence” was created, consisting of group lower (group deviation −2 and −3), group equal (deviation −1, 0 and +1), and group higher (+2 and +3). Then, a repeated measures ANOVA was performed, with the corrected trustworthiness rating change scores as dependent variable, the “average European student” group deviation as within-subjects factor and PPI-group (low vs. high) as between-subjects factor. We also calculated the proportion of conformity (the percentage of trials in which group conflict was followed by conformity) using the corrected rating change estimates and performed a repeated measures ANOVA with proportion conformity as dependent variable, the “average European student” group rating (lower vs. higher) as within-subjects factor and PPI-group as between-subjects factor.

### Data Acquisition

Participants were scanned using a 3.0-Tesla Philips Achieva-scanner at the Leiden University Medical Center. Head motion was restricted using foam inserts surrounding the head. fMRI was performed using T2*-weighted Echo-Planar Images (EPI; TR: 2.2 s, TE: 30 ms, slicematrix 80 × 80, slice thickness: 2.75, FOV: 220 × 220 × 115 mm, slice gap 0.28 mm) in a functional run of 153 volumes. After the functioning scanning, a high resolution T1 structural scan was also acquired (TR: 9.76 ms, TE: 4.59 ms, 140 slices, voxel size: 0.875 mm, FOV: 224 × 177 × 168 mm).

Image analysis was carried out with SPM8 (Welcome Department of Cognitive Neurology, London, UK). The first two volumes of the run were discarded to allow for equilibration of T1 saturation effects and remaining images were realigned to the first volume. For each participant, the images were corrected for differences in slice acquisition time and spatially normalized using the default parameters. The images were corrected for motion, co-registered with the T1 anatomical image and spatially normalized to a T1 template based on the MNI305 stereotaxic space (Cocosco et al., 1997). The normalization algorithm used a 12-parameter affine transformation together with a non-linear transformation involving cosine basic functions and resampled the volumes to 3 mm cubic voxels. Images were spatially smoothed with a Gaussian kernel of 6 mm full-width at half-maximum. Translational movement parameters never exceeded one voxel (<3 mm) in any direction for any subject or scan. The participants who participated had a mean and maximum head movement of 0.08 and 2.52 mm. None of the participants had to be excluded due to excessive head movement.

### fMRI Data Analysis

Statistical analyses were performed on individual participant’s data using the general linear model in SPM8. The fMRI time series data were modeled by a series of events convolved with a canonical hemodynamic response function (HRF). “Social conflict” was modeled as a separate event and was labeled as: Conflict > NoConflict, NoConflict > Conflict; i.e., trials in which individual judgment was in conflict with group opinion vs. trials in which individual judgment was in alignment with the group and reversed. Subsequently, we compared conflict trials followed by conformity vs. conflict trials not followed by conformity and reversed (based on the behavioral results): Conformity > Non-Conformity, and Non-Conformity > Conformity. The duration of the separate events was time-locked with a zero duration. The modeled events based on performed trials were used as covariates of interest in a general linear model along with a basic set of cosine functions that high-pass filtered the data and a covariate for run effects. The least-squares parameter estimates of height of the best-fitting canonical HRF for each condition were used in pairwise contrasts.

Anatomical region of interest (ROI) analyses were performed using a MarsBar toolbox in SPM8 (Brett et al., 2002) to further investigate brain activation for the “Social conflict” and “Conformity” contrasts. We selected anatomical regions based on previous studies (Berns et al., 2005; Klucharev et al., 2009) of the NAc and the amygdala derived from the MarsBaR anatomical toolbox. Additionally, since there is no anatomical RCZ available in the MarsBaR anatomical toolbox, we performed ROI analyses on a 10 mm radius sphere of the RCZ centered on −3, 14, 48 (Klucharev et al., 2009). Beta values reflecting activity were averaged across all voxels in the cluster, resulting in a mean value per ROI for each condition for each participant.

## Results

### Behavioral Results

Total PPI scores ranged between 140 and 181 in the bottom quartile (*N* = 22; *M* = 162.86, SD = 12.76) and from 213–250 for females in the top quartile (*N* = 20; *M* = 229.25, SD = 10.22). An independent-samples *t*-test showed no significant differences in initial trustworthiness ratings between females scoring low (*M* = 4.85, SE = 0.12) or high (*M* = 4.69, SE = 0.12) on psychopathic traits as measured by the PPI, *t*_(40)_ = 0.93, *p* = 0.359 nor between the experimental (*N* = 42, *M* = 4.77, SE = 0.08) and control group (*N* = 32, *M* = 5.07, SE = 0.15), *t*_(51.072)_ = −1.771, *p* = 0.083.

**Figure 2:**
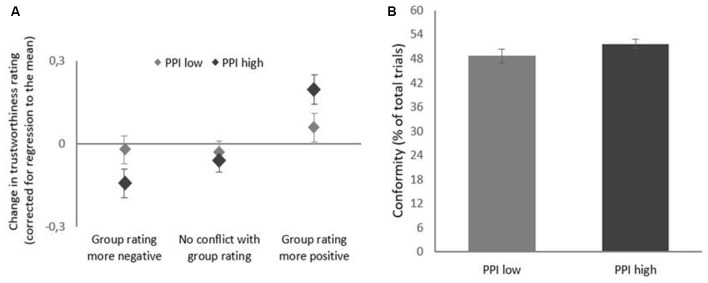
**(A)** Mean behavioral conformity effect for each Psychopathic Personality Inventory (PPI) group, after correction for regression to the mean (RTM). The graph displays the change in trustworthiness ratings following: (1) a more negative (lower trustworthiness) “average European student” group rating compared to individual rating; (2) following no conflict with (same trustworthiness) “average European student” group rating; and (3) following a more positive (higher trustworthiness) “average European student” group rating. Bars indicate standard errors of the mean. **(B)** Mean percentage of conformity for both PPI groups, after correction for RTM.

In line with Schnuerch et al. (2015), a random-coefficients model was fitted to the control group, which revealed that the fixed effect of initial rating was a significant predictor of subsequent rating change (*γ*_10_ = −0533, SE = 0.031, *t*_(32.96)_ = −16.99, *p* < 0.001. The random effect analyses showed that the slopes of initial rating showed little differences between participants ($\sigma_{\delta }^{2}$ = 0.025) The fixed effect coefficient (*γ*_10_) was then used to calculate rating changes scores adjusted for RTM in the experimental group following the formula described by Schnuerch et al. (2015). A repeated measures ANOVA revealed a significant main effect of group deviation on RTM-corrected rating change scores in the experimental group, *F*_(2,80)_ = 8.19, *p* = 0.001, $\eta_{\text{p}}^{2}$ = 0.17, *ε* = 0.95. Pairwise comparisons showed that rating changes were significantly higher when the group had higher trustworthiness ratings (Δ = 0.13, SE = 0.04) compared to when the group rating was lower (Δ = −0.09, SE = 0.04, *p* < 0.001) and compared to trials where the group did not conflict with individual ratings (Δ = −0.05, SE = 0.03, *p* < 0.001). The difference in rating changes between trials with lower group ratings and trials where group ratings were equal to individual ratings did, however, not reach significance (*p* = 0.422). The effect of deviation was not modulated by PPI group, as the interaction of group deviation and PPI score did not reach significance (*p* = 0.084; see Figure 2A).

The rating change scores in the ANOVA used above not only incorporated the occurrence of behavioral conformal adjustments, but also the magnitude of these adjustments. For example, if the trustworthiness of a face is rated with a 3, and the group rated the trustworthiness with a 6, participants can conform to the group by choosing a 4 in the second session, but also by a 5 or 6. When opting for a 5 or 6, rating change score will be larger than when choosing a 4. Thus, the extent to which one adjusts their rating, influences the mean rating change scores. Therefore, we were also interested to see whether the mere occurrence of conformity would differ between PPI groups, regardless of how extreme this conformity-related adjustment was. To this end, we tested whether the total proportion of conformity differed between these groups while taking into account the direction of the group deviation. A repeated measures ANOVA revealed no significant main effect of group deviation (*p* = 0.889) nor any significant interaction effects of group deviation*PPI group (*p* = 0.171). The between-subjects effects was also not significant (*p* = 0.259; see Figure 2B).

### fMRI Results

#### Anatomical ROIs and Sphere

First, we performed a 2 × 2 Mixed ANOVA with Conflict/NoConflict as within-subjects variable, and with Group (Low and High) as between-subjects variable separately for the RCZ, and for the NAc. The results for the RCZ demonstrated no significant main or interaction effects (all *p*’s > 0.12). The results for the left NAc showed no significant main or interaction effects (all *p*’s > 0.13). For the right NAc we did find a significant main effect (*F*_(1,40)_ = 6.50, *p* = 0.015; *η*^2^ = 0.14), demonstrating less deactivation for NoConflict (*M* = −0.24, SD = 0.14) vs. Conflict (*M* = −0.48, SD = 0.14). Yet, neither a main effect for group nor an interaction effect for Conflict/NoConflict*Group was found (*p*’s > 0.41).

**Figure 3:**
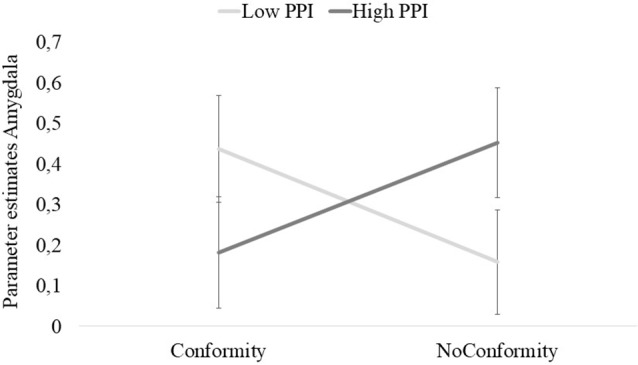
Parameter estimates of the anatomical region of interest (ROI) of the amygdala. The results showed a significant interaction-effect for Conformity*Group (PPI low: females scoring low on psychopathic traits, *N* = 22; PPI high: females scoring high on psychopathic traits, *N* = 20). Females scoring low on psychopathic traits showed a trend in enhanced amygdala activation for conformity relative to non-conformity following conflicts. Additionally, results showed a trend significant between-group-effect for Non-Conformity. Females scoring high on psychopathic traits showed more activation for Non-Conformity compared to the females scoring low on psychopathic traits.

For the amygdala, a 2 × 2 Mixed ANOVA with Conformity/Non-Conformity as within-subjects variable, and Group (Low and High) as between-subjects variable showed an interaction effect for Conformity/Non-Conformity*Group (*F*_(1,40)_ = 5.98, *p* = 0.019; *η*^2^ = 0.13; see Figure 3). We performed pairwise comparisons to test for within and between group differences. The results showed a trend significant within-group-effect for the low scoring group (*p* = 0.081), with higher activation for Conformity (*M* = 0.49, SD = 0.15) vs. Non-Conformity (*M* = 0.17, SD = 0.15). Next, we tested for between group differences, showing a trend significant between-group-effect for Non-Conformity (*p* = 0.078). Females scoring high on psychopathic traits showed more activation for Non-Conformity (*M* = 0.49, SD = 0.15) compared to the females scoring low on psychopathic traits (*M* = 0.25, SD = 0.16).

## Discussion

The current study was the first to investigate how individual differences in psychopathic traits in females are associated with the neural mechanisms involved in social (non)conformity. We used an established social conformity paradigm to detect conformity to group opinion and to investigate associated neural processing of group opinion (Berns et al., 2005; Klucharev et al., 2009; Campbell-Meiklejohn et al., 2010, 2012; Nook and Zaki, 2015). First, our behavioral results show that conformity behavior does not differ between females scoring low and high on psychopathic traits. Second, neuroimaging results showed that social conflict did not activate the RCZ in either group, whereas alignment activated the NAc similarly in both groups. Third, we found that the amygdala was differently involved for conflict trials that were followed by conformity or non-conformity depending on the group: females scoring low on psychopathic traits tended to show higher amygdala activation for conformity relative to non-conformity following conflicts, whereas females scoring high on psychopathic traits showed higher activation than the low scoring group when conflicting feedback resulted in not conforming. Overall, this study partly replicates previous findings of Klucharev et al. (2009) and Berns et al. (2005) but also extends these outcomes by showing activation patterns that seem to be dependent on the level of psychopathic traits.

Our behavioral results showed that the groups showed no differences in conformity behavior. Numerically, females scoring high on psychopathic traits even seemed to conform to a greater extent compared to the females scoring low. These results contradict our initial hypothesis. Based on evidence for norm-violating behavior in psychopaths and reduced concern for others in individuals scoring high on psychopathic traits, we expected that conformity to a normative group opinion would be decreased in females with high levels of self-reported psychopathic traits (Kiehl and Hoffman, 2011; Seara-Cardoso et al., 2012; Foulkes et al., 2014b). However, our results suggest that these females show typical conformity behavior. In interpreting this finding, it is important to note that our participants were high-functioning university students. These students are considered “successful” within society, which could be explained by intact or even enhanced neurobiological and cognitive functioning. This allows them to achieve goals using more covert and nonviolent methods (Gao and Raine, 2010). In line with this, several experimental studies indicate that individuals in the general population do not possess the same behavioral deficits that characterize the clinical population in a range of social and emotional tasks (Gordon et al., 2004; Glenn et al., 2009; Marsh et al., 2011; Vieira et al., 2014). Yet, some experimental studies in the general population do indicate alterations in social behavior in relation to psychopathic traits (Rilling et al., 2007; Curry et al., 2011). For example, Rilling et al. (2007) reported that healthy participants with high levels of psychopathic traits defected more often and were less likely to continue cooperating after establishing mutual cooperation with a partner in a prisoner’s dilemma game, but this effect was significant only in male participants. In favor of this notion, it has been argued that gender and societal factors may affect the expression of psychopathic traits (Forouzan and Cooke, 2005; Kreis and Cooke, 2011). For example, females are generally more fearful and risk-averse, and have better social skills. In contrast, males are usually more assertive and fearless compared to females (Kreis and Cooke, 2011). This suggests that typical traits associated with psychopathy such as reduced interpersonal concern might be less prominent in females. Moreover, gender roles and societal expectations might also shape differences in behavior. For example, whereas the masculine gender roles endorse being independent, dominant and assertive, the feminine gender roles promote passivity, compliance and conformity, as well as the expression of empathy (Block, 1983; Blashill, 2011). Females might benefit more from subtle techniques to attain their goals, and therefore can be expected to show enhanced submissive and adaptive behavior including conformity. It has also been suggested that psychopathic females use these stereotypical female traits as a manipulative facade to exploit others using more subtle interpersonal strategies (Kreis and Cooke, 2011). Another explanation for the lacking difference in behavior could be related to the different underlying motivations in females scoring low vs. females scoring high on psychopathic traits. Females scoring low on psychopathic traits could be motivated by a desire for social approval leading to feelings of belongingness, whereas females scoring high on psychopathic traits could be motivated by a desire for manipulation or by doing what’s right in order to prevent to be conspicuous (Cialdini and Goldstein, 2004; Foulkes et al., 2014a). An alternative explanation, apart from gender, could be that some inventories might be more sensitive for psychopathy than others, which could explain differential findings between males and females. Taken together, female psychopathic traits seem to be less apparent on the behavioral level, which may be due to gender, societal factors, psychopathy inventories, and different underlying motivations. Future research on psychopathic traits and social conformity should, therefore, focus on direct comparisons between the female and male population using the same psychopathy inventories, and on inward beliefs.

Imaging findings of the social conflict contrast showed that for both groups, conflict with group opinion did not activate the RCZ differently compared to no conflict, whereas no conflict or alignment with the group activated the NAc. These results are therefore only partly comparable with the results of Klucharev et al. (2009). In contrast with their study, conflict with group opinion did not activate the RCZ. Additionally, in contrast with Klucharev et al. (2009), we observed NAc activation during social alignment (no conflict) rather than NAc deactivation during social conflict. In agreement with our findings, other studies on social conformity have also found activation of the NAc during social alignment with group opinion rather than deactivation during social conflict (Campbell-Meiklejohn et al., 2010; Nook and Zaki, 2015). NAc activation during social alignment is thought to reflect the rewarding value of being in alignment with the opinion of others, and as such, could reflect a positive (social) prediction error (Campbell-Meiklejohn et al., 2010). Prior studies investigating social prediction errors also found an important role for the NAc. For example the study by Jones et al. (2011), reporting that the striatum plays an important role in positive social prediction errors by updating social expectations in order to adapt to changing environments. An important role for the NAc in social learning through prediction errors has also been shown in the study by Jarcho et al. (2015), who investigated social prediction errors in socially anxious vs. non-socially anxious adolescents and adults while receiving positive or negative feedback from peers they were not interested to chat with (low-value peers) and peers they were interested to chat with (high-value peers). The results showed that specifically in socially anxious adolescents, unexpected positive feedback from high-valued peers corresponded to heightened striatal activity and a failure to recall the positive feedback. Although we did not investigate social anxiety in our sample, the study by Jarcho et al. (2015) shows that how we value the other party can influence the saliency of our neural network. Therefore, it would be interesting to include this factor in future studies investigating psychopathic traits in order to disentangle the complex (neural) social learning mechanisms.

The fact that we did not find group differences regarding the social conflict contrast suggests that females high on psychopathic traits might not be characterized by the neural impairments in prediction error signaling that have previously been observed in the mainly (clinical) male population. This appears consistent with the behavioral results that showed intact conformity behavior in females high in psychopathic traits. According to the reinforcement learning account of social conformity (e.g., Klucharev et al., 2009), the prediction-error related signals in the RCZ and Nac indicate the need for behavioral adjustment, and as such, should serve to reinforce conformity behavior. If activity in these areas indeed predicts subsequent conformity, then activity should be stronger for trials in which social conflict was followed by conformity. However, when comparing conflict trials followed by conformity vs. no conformity, we did not find enhanced RCZ and Nac (de)activation in conformity trials. Therefore, the data do not seem to support the notion that larger RCZ and NAc responses may lead to more conformity. Notably, several other studies did not find the expected correlations between the behavioral and neural effects in the social conformity paradigm either (Kim et al., 2012; Shestakova et al., 2013; Huang et al., 2014). Using facial judgment tasks similar to the task we employed, these EEG studies showed that the conflict with group opinion triggered prediction error-signals (FRN), yet no relation between these components and conformity behavior was obtained. Therefore, we need more research in order to get a better understanding of other factors involved in the detection of social conflict and the subsequent behavioral change.

Next, our results showed that conflicts followed by (non)conformity were associated with amygdala activation. The follow-up analyses revealed trend-significant effects, suggesting that conflicts followed by conformity showed similar amygdala activation in both groups, whereas conflicts followed by non-conformity was associated with higher amygdala activation in the high scoring females. Although we are cautious in interpreting this outcome, it is remarkably in line with repeatedly demonstrated distorted amygdala activation in individuals scoring high on psychopathic traits when studying non-social aversive learning, suggesting altered emotional salience of experiencing a social conflict (e.g., Birbaumer et al., 2005; Schultz et al., 2016). A possible explanation for this could be that females with high levels of psychopathic traits attribute higher salience (as indicated by enhanced amygdala activation) to those conflicts that were followed by non-conformity compared to conflicts followed by conformity. As the amygdala is thought to play an important role in stimulus-reinforcement learning, and particularly aversive learning (Blair, 2007), this activity pattern seems counterintuitive. From an aversive learning perspective, enhanced salience or aversiveness of conflicts as indicated by increased amygdala activation should serve to adapt behavior as to avoid these conflicts in the future, and thus stimulate conformity rather than non-conformity. As such, the higher amygdala activation observed in high scoring females might be dysfunctional, as increased activity in this area seems to interfere with making the most adaptive choice, namely conformity. Additionally, it should be noted that when contrasting conformity vs. non-conformity, the low scoring group showed a tendency for higher amygdala activation. The higher amygdala activation observed in the low scoring females fits with prior studies including healthy individuals, as higher activity in this region is indicative for conformity behavior (Berns et al., 2005).

We speculate that the between-group pattern of amygdala activation might be explained by the concept of “memory conformity,” which has been explained as a change of memory by social influence. According to the social psychology literature, conformity can be separated into two forms: (1) private conformity: conforming to a group norm, leading to (long-term) altered persistent memory errors; and (2) public conformity: conforming to a group norm, while inwardly remaining convinced of own memories and beliefs (Wright et al., 2009). Edelson et al. (2011) investigated the role of the amygdala in “memory conformity” in a social context, using a protocol in order to test for the persistence of memory errors following social manipulation. First, participants performed a memory test individually from which the correct trials were selected in order to use them in the second social manipulation test. Before performing the second test themselves, they observed four co-participants performing the task in which the co-participants, unknown to the participant, structurally gave false answers. Finally, conformity behavior was tested by measuring persistent memory errors while participants performed the same memory test later in time, without the social manipulation. Results of the study of Edelson et al. (2011) showed enhanced amygdala activation when participants showed persistent memory errors, specifically after the social manipulation. Overall, these results indicate that the memory of participants was altered by social influence (i.e., private conformity). This finding is in line with the outcomes of our study as females scoring low on psychopathic traits demonstrated a tendency for heightened amygdala activity when conforming to the group following a conflict. Since the amygdala plays an important role in persistent memory errors following social manipulation, this specific outcome in the low scoring group suggests similar private conformity behavior compared to the findings of Edelson et al. (2011). Additionally, our results showed a trend significant group effect for non-conformity, with females scoring high on psychopathic traits showing more activation for non-conformity compared to females scoring low on psychopathic traits. The enhanced amygdala activity in the high scoring females, when not conforming to the group norm, might suggest that they only publicy conformed to the group norm, an interpretation that is obviously in need of future investigation. Therefore, we again would like to emphasize that we are cautious in interpreting these results, as the follow-up analyses of the significant interaction only revealed trend-significant effects.

The current study also holds some limitations. First, although we included enough participants to compare groups on a neural level, on a behavioral level the groups are rather small to make a sufficient comparison. Future studies should further investigate whether higher levels of psychopathic traits are of influence regarding conformity behavior while taking into account the possibility that individuals scoring high on psychopathic traits might over-conform as was suggested by the trend significant effect in the current study. Second, we created groups based on the total scores on the PPI-SF (Tonnaer et al., 2013), which limits the opportunity to test for sub-dimensions. We know from previous studies that psychopathy is a multidimensional construct (Lilienfeld, 2018), which also shows different profiles for males and females (Cale and Lilienfeld, 2002). As such, it might be worthwhile for future studies to include larger samples and to investigate the neural correlates of these distinct psychopathic subtypes in females using a dimensional approach. Moreover, participants experienced a social conflict in 67% of trials, which could have led to conflict habituation resulting in the absent RCZ main effect for conflict vs. no conflict. This is also in line with prior studies (e.g., Braver et al., 2001) who found that conflict-related brain responses are particularly enhanced if the conflict occurs infrequently (e.g., in 20% of the trials). Therefore, future studies might benefit from using a lower conflict frequency combined with more trials in order to create extra power to analyze conflict level and valence. Lastly, we did not account for female hormonal status as a possible confounding factor. Since we included an all-female sample, and prior studies have found oral contraceptives to influence amygdala and salience resting-state network (Petersen and Cahill, 2015; Engman et al., 2018), future studies should take this into account.

In summary, our results showed no behavioral differences in conformity to a normative group opinion in a sample of high-functioning females scoring low or high on psychopathic traits. Additionally, fMRI results showed no RCZ activity in both groups in case their opinion was conflicting with the opinion of the group, contrary to the findings of Klucharev et al. (2009). In case of no conflict, both groups showed reward-related activity in the NAc suggesting the involvement of (social) reward processes or social prediction errors when being in alignment with the group. Finally, we observed differential brain patterns for both groups in the amygdala during social conflict with group opinion, specifically related to (non)conformity behavior. We speculate that this might suggest that dependent on the level of psychopathic traits people used distinct neural mechanisms in order to achieve similar behavioral outcomes, possibly reflecting altered emotional salience of experiencing social conflict. Our findings emphasize the need to further explore the role of individual differences in social conformity, especially since the effects are rather small and only tested in relatively small groups. However, our sample was unique in its focus on psychopathic traits in an all-female sample. Gaining more insights into psychopathic traits in females is important, as it might have implications for the diagnosis and treatment of psychopathic traits in women (Wynn et al., 2012). Future studies should further investigate alterations in the neural mechanisms of social conformity, not only in females, but also in the male and clinical population. Additionally, future studies should collect data on how conformity is experienced. Perhaps individuals with high levels of psychopathic traits do not experience non-conformity as a social aversive learning signal. In that case, conforming to group norms might only be a strategy to successfully adapt to uncertain circumstances for the females scoring high on psychopathic traits, whereas the low scoring females might be predominantly motivated by a desire for social approval. Moreover, it would also be interesting to focus on whether individuals scoring high on psychopathic traits publically conform to group norms in order to be able to successfully adapt to uncertain circumstances or out of a desire for social approval, possibly reflecting a discrepancy between conformity behavior and inward beliefs. Such investigations could provide us with broader insights into the behavioral and neural anomalies associated with psychopathic traits, as well as potential gender differences. To conclude, the current study takes a first step in investigating individual differences in adaptive behavior when facing uncertain social situations and the neural mechanisms involved in this process.

## Ethics Statement

This study was carried out in accordance with the recommendations of the Institutional Review Board of the Leiden University Medical Center with written informed consent from all subjects. All subjects gave written informed consent in accordance with the Declaration of Helsinki. The protocol was approved by the Institutional Review Board of the Leiden University Medical Center.

## Author Contributions

SO, MJ, and NK collected the data. SO, MJ, NK, and EB analyzed the data. SO, MJ, and EB wrote the manuscript, provided feedback, and revised the manuscript. All authors approved the final version.

## Conflict of Interest Statement

The authors declare that the research was conducted in the absence of any commercial or financial relationships that could be construed as a potential conflict of interest.
